# Supplementary material for: Multi-signal regulation of the GSK-3β homolog Rim11 controls meiosis entry in budding yeast
Source: EMBO J. 2024 Jun 17;43(15):3256–86. doi: 10.1038/s44318-024-00149-7 (PMC11294583; doi:10.1038/s44318-024-00149-7)
Supplement: Supplementary file 1 — Table EV1 [file 44318_2024_149_MOESM1_ESM.docx]

**Table EV1. Oligo nucleotide sequences used**

| name | sequence | gene | notes |
| --- | --- | --- | --- |
| \| JK_IME2_qRT_F \| \| --- \| | CAGATTTTGGTTTGGCACGC | IME2 |  |
| JK_IME2_qRT_R | TACAGTAACTTCCACCGCCA | IME2 |  |
| FvWACT1Frt | gtaccaccatgttcccaggtatt | ACT1 |  |
| FvWACT1Rrt | caagatagaaccaccaatccaga | ACT1 |  |
| pUB415 | TGCCTCTTTAGGCGATTCGT | IME2 | Figure 6E, Appendix Figure S5 |
| pUB416 | GCTCGAACTTTTCCCGTGATT3 | IME2 | Figure 6E, Appendix Figure S5 |
| pUB2598 | GTACCACCATGTTCCCAGGTATT3 | ACT1 | Figure 6E, Appendix Figure S5 |
| pUB2599 | AGATGGACCACTTTCGTCGT3 | ACT1 | Figure 6E, Appendix Figure S5 |
