# Supplementary material for: Multi-signal regulation of the GSK-3β homolog Rim11 controls meiosis entry in budding yeast
Source: EMBO J. 2024 Jun 17;43(15):3256–86. doi: 10.1038/s44318-024-00149-7 (PMC11294583; doi:10.1038/s44318-024-00149-7)
Supplement: Supplementary file 2 — Table EV2 [file 44318_2024_149_MOESM2_ESM.docx]

**Table EV2. Plasmids used in this study**

| Plasmid No. | Name |
| --- | --- |
| \| FW_P770 \| \| --- \| | pNH605-RIM11-mNG |
| FW_P772 | pNH605-rim11-3SA-mNG |
| FW_P779 | pNH605-rim11-Y199F­-mNG |
| FW_P780 | pNH605-RIM11_NLS-mNG |
| FW_P730 | pNH604-pIME1-sfGFP-IME1-L321F |
| FW_P506 | pNH604-pIME1-sfGFP-IME1 |
