# Supplementary material for: Multi-signal regulation of the GSK-3β homolog Rim11 controls meiosis entry in budding yeast
Source: EMBO J. 2024 Jun 17;43(15):3256–86. doi: 10.1038/s44318-024-00149-7 (PMC11294583; doi:10.1038/s44318-024-00149-7)
Supplement: Supplementary file 3 — Appendix [file 44318_2024_149_MOESM3_ESM.pdf]

Appendix for:

Multi-signal regulation of the GSK-3β homolog Rim11 controls meiosis entry in budding yeast

Table of Contents:

Page 2, 3: Appendix Figure S1

Page 4: Appendix Figure S2

Page 5: Appendix Figure S3

Page 6, 7: Appendix Figure S4

Page 8, 9: Appendix Figure S5

Appendix Figure S1

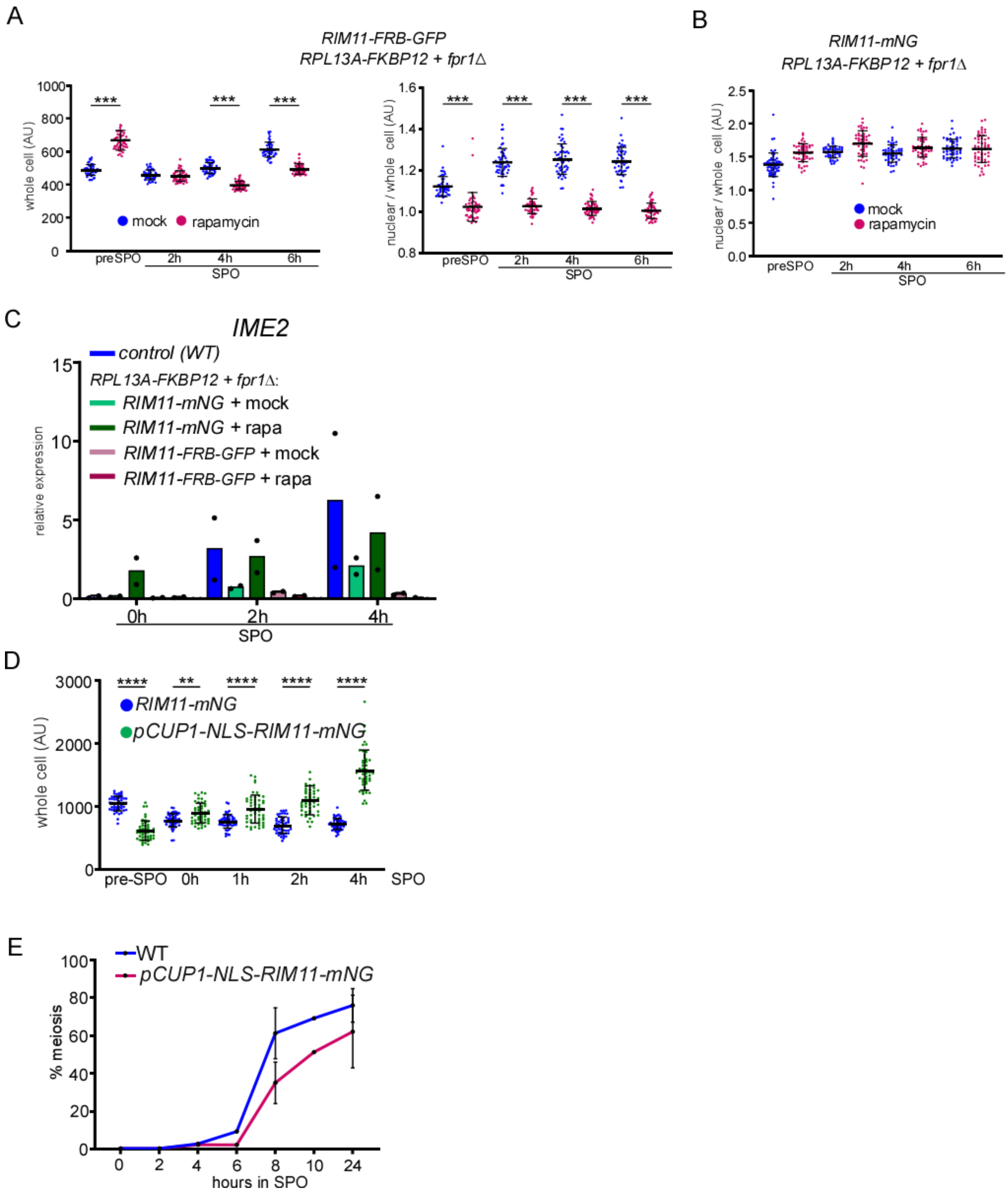

Appendix Figure S1. Rim11 nuclear localization is required for meiosis, but not sufficient

(**A**) Cells harboured *RIM11-FRB-GFP*, *RPL13A-FKBP12* and *fpr1*Δ. Quantification of whole cell signal (left) and nuclear over whole cell signal (right) of Rim11-FRB-GFP in either mock treated or treated with rapamycin (FW11124) in pre-sporulation medium (pre-SPO), and different time points in sporulation medium (SPO). At least 50 cells were per time pointed were quantified. Black bars with error bars represent the mean+SD. One-way ANOVA; *** $P<0.001$. (**B**) Nuclear over whole cell signals for the Rim11-mNG in cells harbouring *RPL13A-FKBP12* and *fpr1*Δ (FW11126). Black bars with error bars represent the mean+SD. **(C)** *IME2* expression in WT cells (1511), and in cells harbouring *RPL13A-FKBP12* and *fpr1*Δ (FW11257) by itself or together with *RIM11-FRB-GFP* (FW11124) or *RIM11-mNG* (FW11126) induced in enter meiosis. Samples were taken at the indicated time points, RT-qPCR was performed to determine *IME2* expression. The relative signals normalized over *ACT1* of n=2 biological repeats are shown. **(D)** Whole-cell signal (left) and nuclear over whole cell signal (right) signal of WT Rim11-mNG (FW10776) and Rim11-mNG fused to NLS under control of *CUP1* promoter (FW11206) in cells induced to enter meiosis. One-way ANOVA; ** $P<0.01$. **** $P<0.0001$. **(E)** Onset of meiosis in WT (FW1511) and *pCUP1-NLS-RIM11*-mNG (FW11206). Cells were induced to sporulate, and samples were taken at the indicated time points, fixed, and stained with DAPI. Cells that contained two more DAPI masses were considered to have entered meiosis. Error bar repressed mean of n=3 +/-SEM.

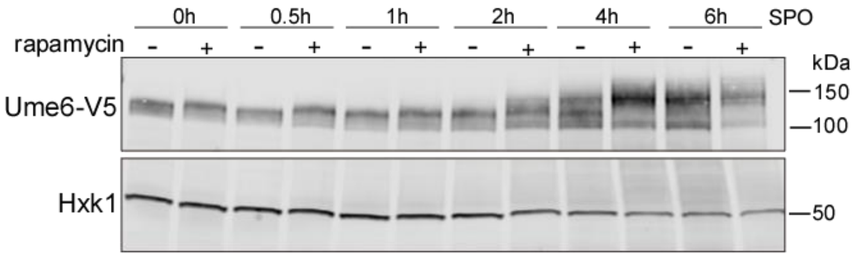

Appendix Figure S2. TORC1 and PKA control Rim11 localization and expression

Ume6 migration as determined by western blotting (FW1208). Cells were induced to enter meiosis and were either untreated or treated with rapamycin. Western blot membrane was also probed anti-Hxk1 antibodies as a loading control Hxk1.

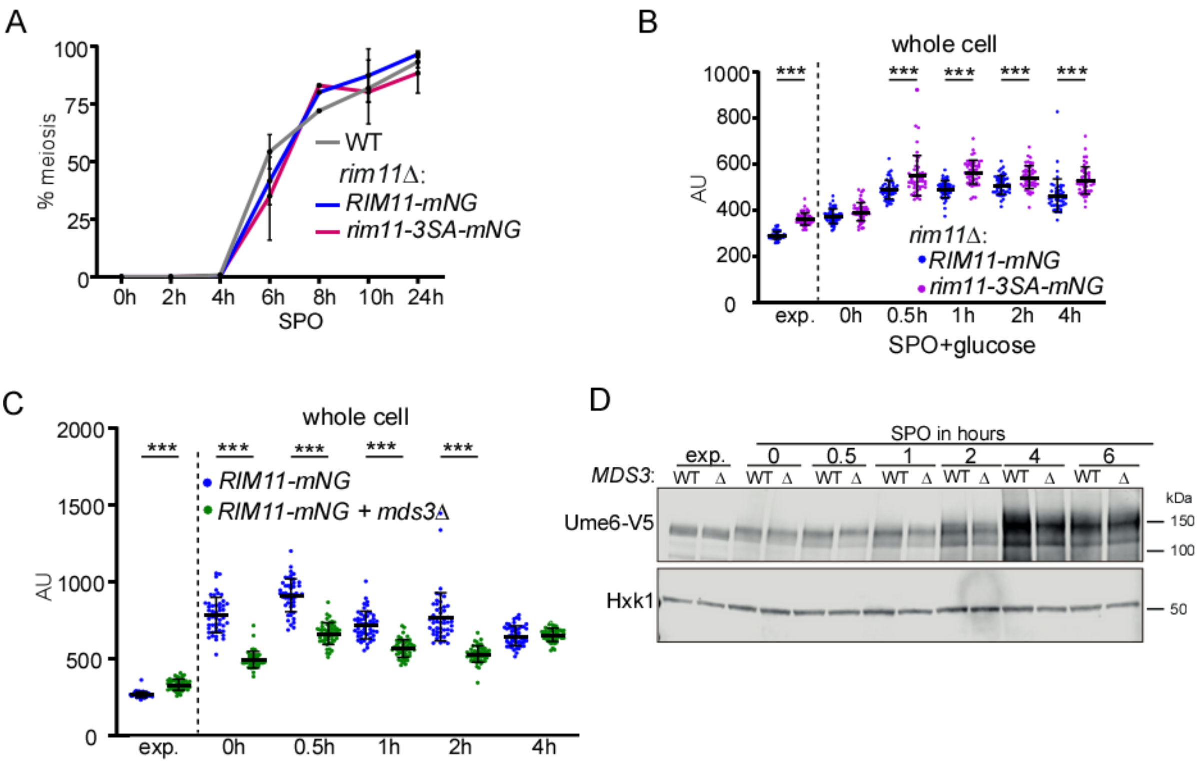

Appendix Figure S3. PKA and TORC1 control Rim11 via distinct mechanisms

(A) Onset of meiosis in WT cells and cells harbouring *rim11Δ* expressing an integration plasmid harbouring *RIM11-mNG* and *rim11-3SA-mNG* (FW1511, FW10776 and FW10778). The mean signals of n=3 + SEM of biological repeats are shown. **(B)** Whole cell quantification of Rim11-mNG and rim11-3SA-mNG in SPO + 1% glucose. Black bars with error bars represent the mean+SD. One-way ANOVA; ***P<0.001. **(C)** Whole-cell quantification of Rim11-mNG in control and in *mds3Δ* cells grown till the exponential phase and induced to enter meiosis. Black bars with error bars represent the mean+SD. One-way ANOVA; ***P<0.001. **(D)** Ume6-V5 migration as determined by western blotting in WT and *mds3Δ*. Cells were grown till the exponential phase and induced to enter meiosis. Samples were taken at the indicated time points. Membranes were probed with anti-V5 antibodies and Hxk1 antibodies as a loading control (FW1208, FW11251).

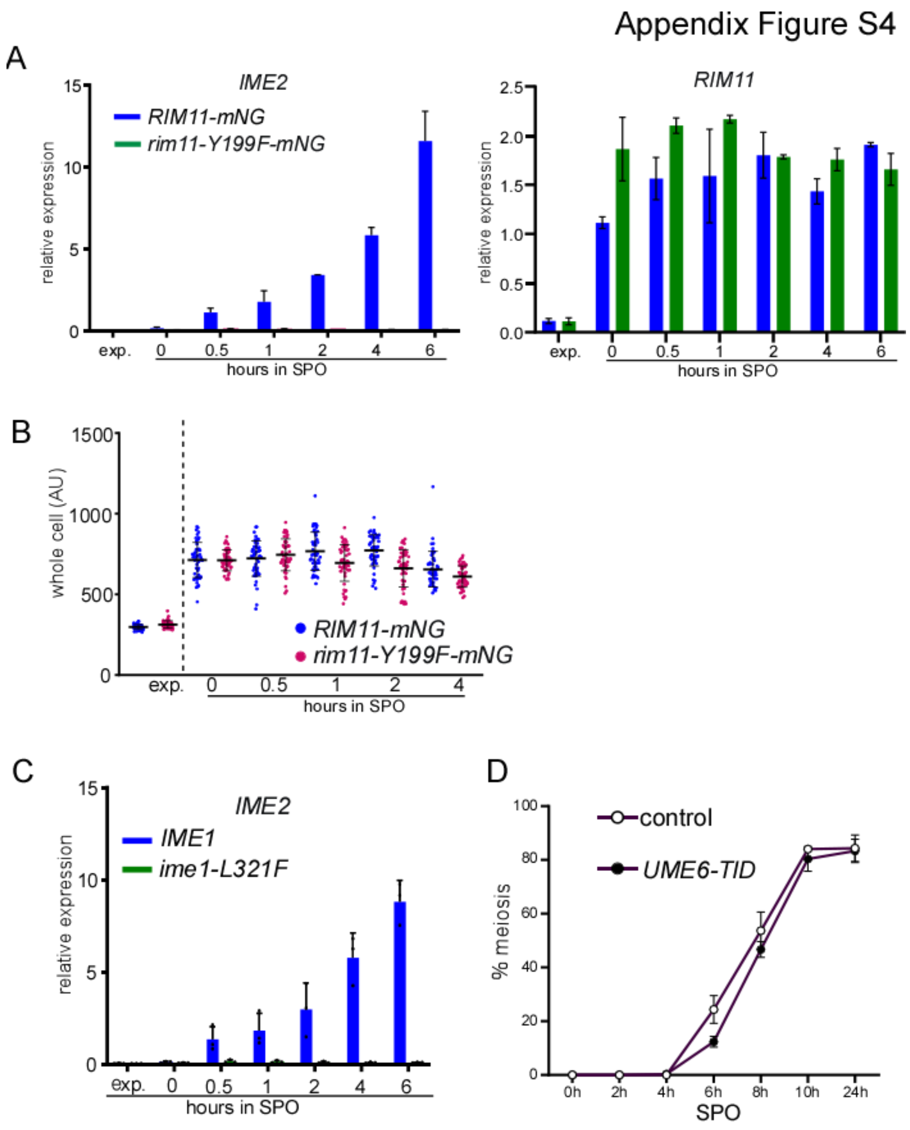

Appendix Figure S4. Ime1 is essential for Rim11 directed Ume6 phosphorylation

(A) *IME2* and *RIM11* mRNA expression in cells harbouring *rim11*∆ expressing an integration plasmid harbouring *RIM11-mNG* or *rim11-Y199F-mNG* (FW10776, FW10983) cells induced to enter meiosis. mRNA expression levels were normalized to *ACT1*. The mean value of n=3 biological repeats is shown. **(B)** Rim11-mNG whole cell localization in cells harbouring *rim11*∆ expressing an integration plasmid harbouring *RIM11-mNG* or *rim11-Y199F-mNG* (FW10776, FW10983). Black bars with error bars represent the mean+SD. **(C)** *IME2* expression in *ime1*∆ cells

harbouring an integration plasmid expressing *sfGFP-IME1* or *sfGFP-ime1-L321F* (FW11231, FW11233). *IME2* expression was normalized to *ACT1*. The mean value of n=3 biological repeats is shown. **(D)** Onset of meiosis in WT (control, FW1511) and *UME6-TID* (FW11422). Samples were taken at the indicated time points, fixed, and stained with DAPI. Cells that contained two more DAPI masses were considered to have entered meiosis. The mean signals of n=3 + SEM biological repeats are shown.

Appendix Figure S5

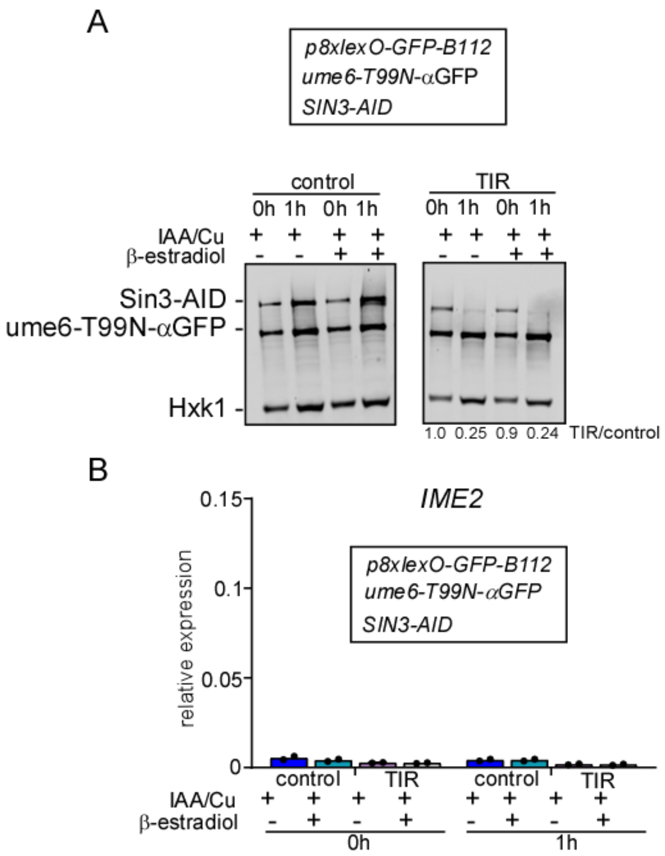

Appendix Figure S5. Rewiring of Ume6 regulon makes Rim11 and Ime1 partly dispensable

(A) Sin3 depletion determined by western blot. Cells expressing *ume6-T99N-αGFP* and *GFP-B112* under control of eight lexO sites (*p8xlexO-GFP-B112*) and *SIN3-AID* were untreated or treated with β-estradiol in the absence (control) or presence (TIR) of an inducible TIR1 ligase (*pCUP1-OsTIR1*) construct (UB36872, UB36874). All cells were treated with 3-indoleacetic acid (IAA) and copper sulphate to induce TIR1. Western blots were probed with anti-V5 antibodies to detect Sin3-AID and ume6-T99N-αGFP. As a loading control, Hxk1 was used. The efficiency of Sin3-AID depletion was assessed by calculating the relative Sin3-AID levels compared to Hxk1 and then determining the ratio of TIR to control. **(B)** *IME2* mRNA expression in

cells grown in the exponential growth phase. Same strains as in A. *IME2* expression normalized by *ACT1*. The mean signal of n=2 biological repeats is shown.
