# Supplementary material for: Multi-signal regulation of the GSK-3β homolog Rim11 controls meiosis entry in budding yeast
Source: EMBO J. 2024 Jun 17;43(15):3256–86. doi: 10.1038/s44318-024-00149-7 (PMC11294583; doi:10.1038/s44318-024-00149-7)
Supplement: Supplementary file 7 — Movie EV1 [file 44318_2024_149_MOESM7_ESM.zip › Kociemba_Movie EV1 legend.docx]

**Movie EV1.** Movies EV1 display meiosis entry in an asynchronous population of cells bearing Htb1-mCherry and Rim11-mNG (FW10297). The movie was made with uncompressed file frames obtained using the microscopy and microfluidics setup described in the materials and methods. Each channel was corrected for contrast and brightness to ease the visualization of nuclear signals. Plots represent the quantification of the background-corrected and spectrally-unmixed raw time series of the cell outlined in yellow. Pauses are included to highlight landmark events such as nuclear division or peaks in nuclear accumulation of relevant proteins. Plots advance according to the frame rate. Sampling rate = 12 min. Playback = 5 frames per second.
