# Supplementary material for: Multi-signal regulation of the GSK-3β homolog Rim11 controls meiosis entry in budding yeast
Source: EMBO J. 2024 Jun 17;43(15):3256–86. doi: 10.1038/s44318-024-00149-7 (PMC11294583; doi:10.1038/s44318-024-00149-7)
Supplement: Supplementary file 8 — Movie EV2 [file 44318_2024_149_MOESM8_ESM.zip › Kociemba_Movie EV2 legend.docx]

**Movie EV2.** Movies EV2 display meiosis entry in an asynchronous population of cells bearing fluorescently labeled proteins Rim11- mKOκ, Ume6-TFP, and sfGFP-Ime1 (FW11243). The movie was made with uncompressed file frames obtained using the microscopy and microfluidics setup described in the materials and methods. Each channel was corrected for contrast and brightness to ease the visualization of nuclear signals. Plots represent the quantification of the background-corrected and spectrally-unmixed raw time series of the cell outlined in yellow. Deliberate pauses are included to highlight landmark events such as nuclear division or peaks in nuclear accumulation of relevant proteins. Plots advance according to the frame rate. Sampling rate = 12 min. Playback = 5 frames per second.
