# Supplementary figures and images for: Multi-signal regulation of the GSK-3β homolog Rim11 controls meiosis entry in budding yeast
Source: EMBO J. 2024 Jun 17;43(15):3256–86. doi: 10.1038/s44318-024-00149-7 (PMC11294583; doi:10.1038/s44318-024-00149-7)

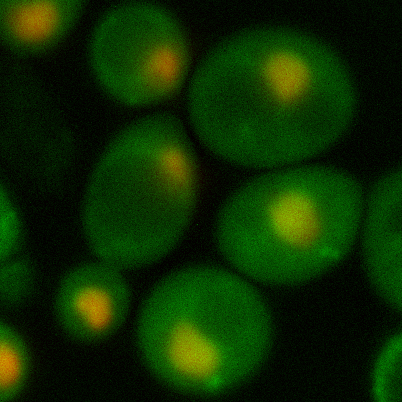

Supplement: Supplementary file 10 — Source data Fig. 2 [file 44318_2024_149_MOESM10_ESM.zip › Figure 2/2B. -rap merged cropped.tif]

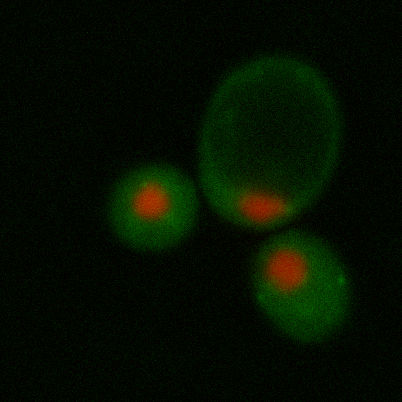

Supplement: Supplementary file 10 — Source data Fig. 2 [file 44318_2024_149_MOESM10_ESM.zip › Figure 2/2B. merged cropped.tif]

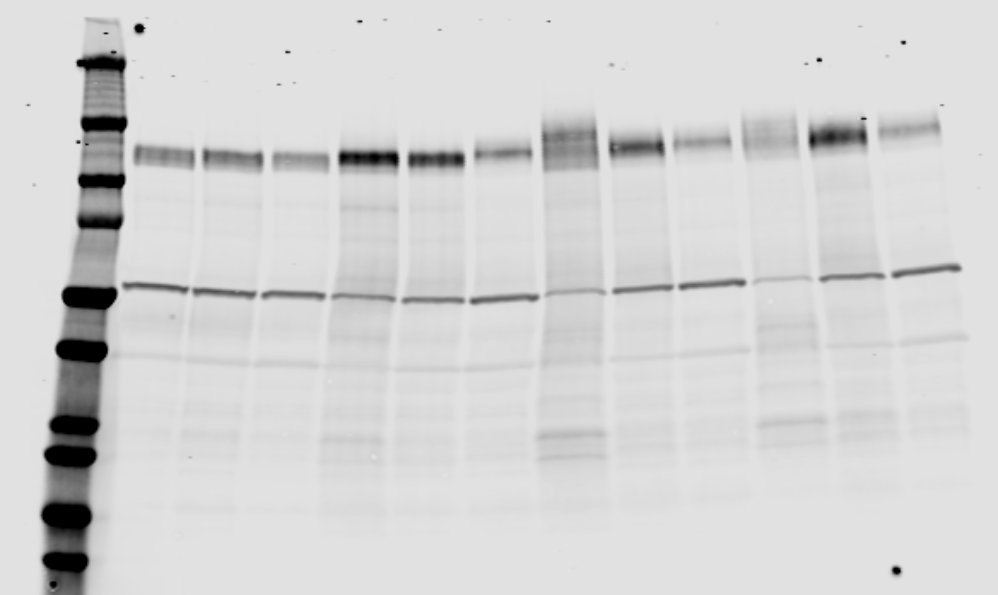

Supplement: Supplementary file 10 — Source data Fig. 2 [file 44318_2024_149_MOESM10_ESM.zip › Figure 2/2F. Uncropped WB.tif]

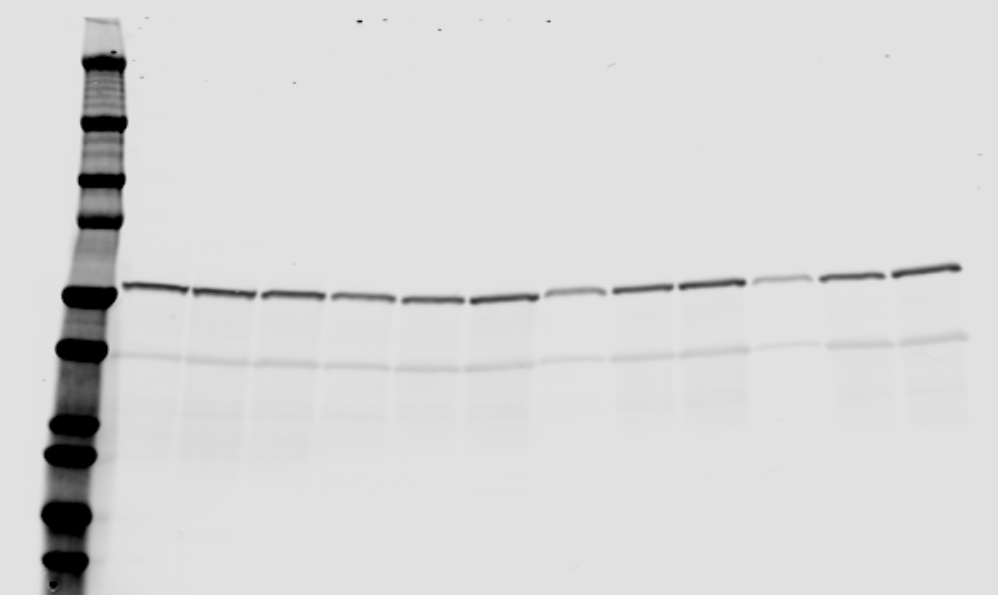

Supplement: Supplementary file 10 — Source data Fig. 2 [file 44318_2024_149_MOESM10_ESM.zip › Figure 2/2F. Uncroppped WB Hxk1.tif]

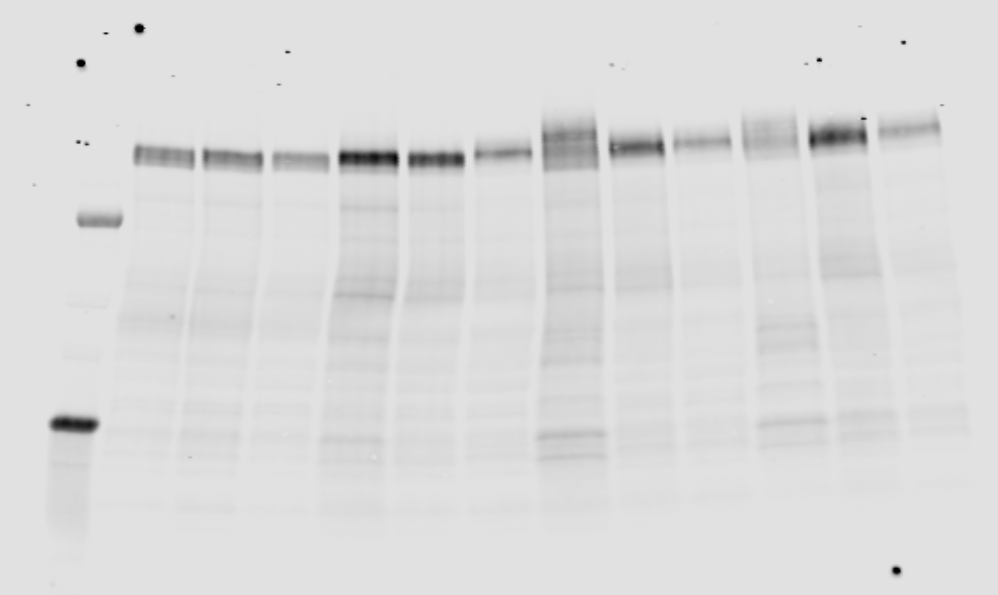

Supplement: Supplementary file 10 — Source data Fig. 2 [file 44318_2024_149_MOESM10_ESM.zip › Figure 2/2F. Uncroppped WB Ume6.tif]

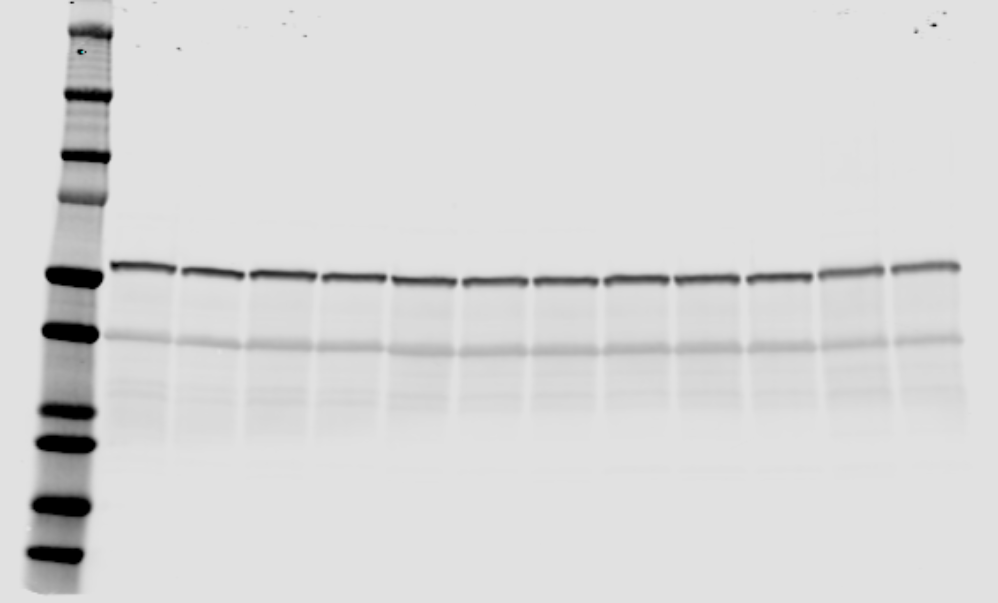

Supplement: Supplementary file 10 — Source data Fig. 2 [file 44318_2024_149_MOESM10_ESM.zip › Figure 2/2G. Uncropped WB Hxk1.tif]

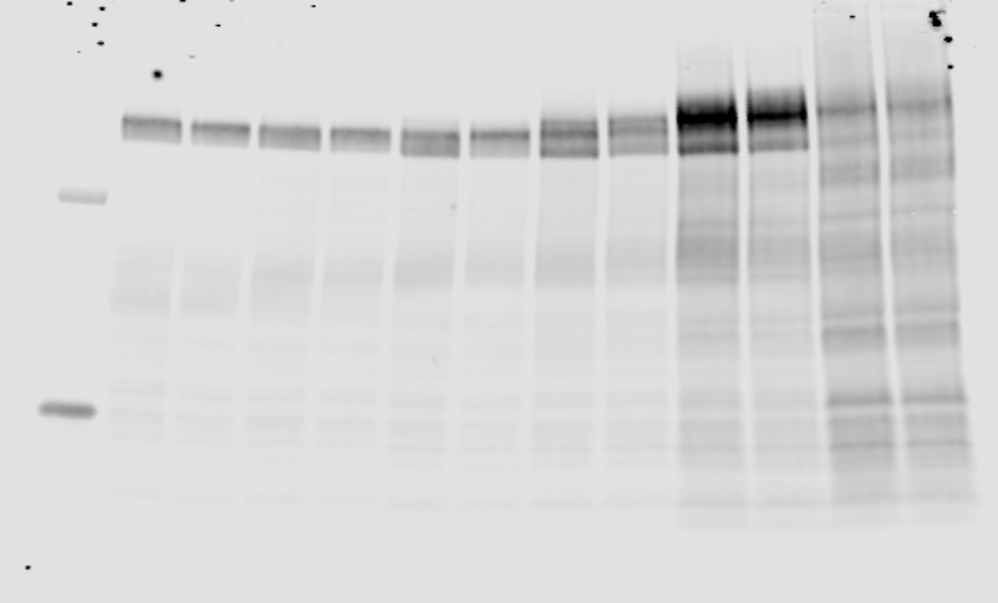

Supplement: Supplementary file 10 — Source data Fig. 2 [file 44318_2024_149_MOESM10_ESM.zip › Figure 2/2G. Uncropped WB Ume6.tif]

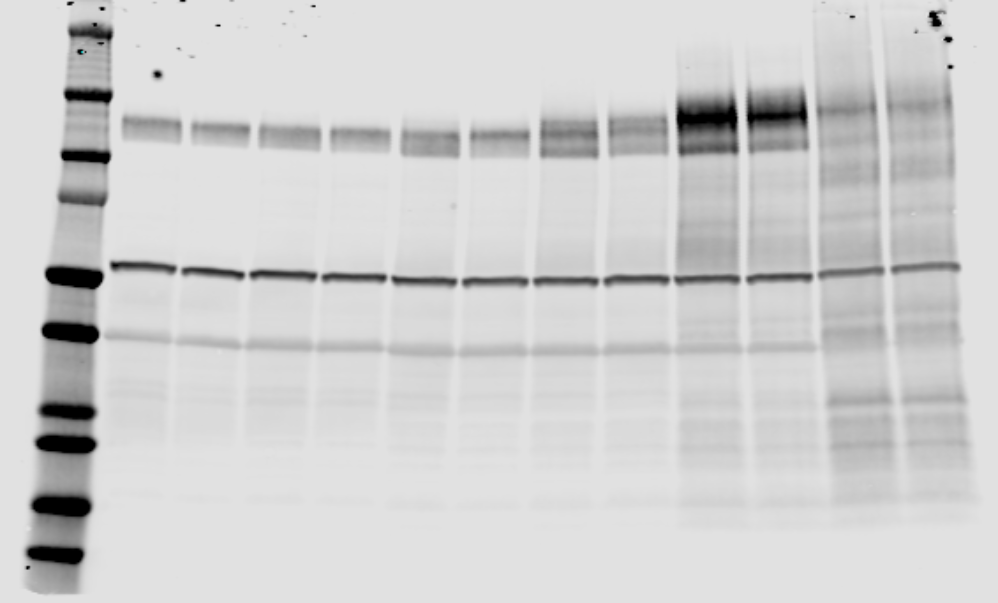

Supplement: Supplementary file 10 — Source data Fig. 2 [file 44318_2024_149_MOESM10_ESM.zip › Figure 2/2G. Uncropped WB.tif]

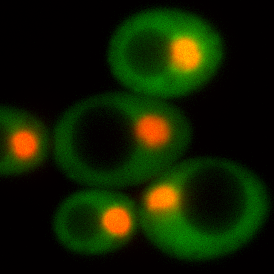

Supplement: Supplementary file 11 — Source data Fig. 3 [file 44318_2024_149_MOESM11_ESM.zip › Figure 3/3A. YP merged cropped.tif]

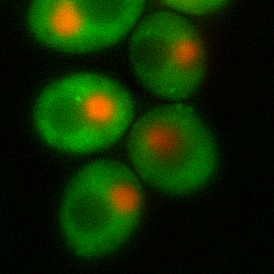

Supplement: Supplementary file 11 — Source data Fig. 3 [file 44318_2024_149_MOESM11_ESM.zip › Figure 3/3A. YPD merged cropped.tif]

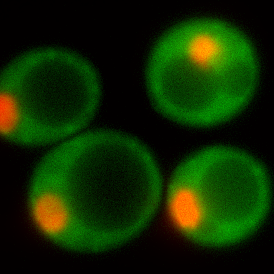

Supplement: Supplementary file 11 — Source data Fig. 3 [file 44318_2024_149_MOESM11_ESM.zip › Figure 3/3A. YPD+rapa merged cropped.tif]

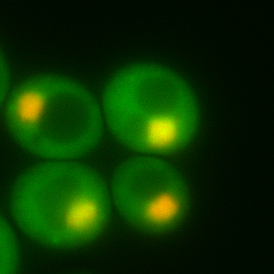

Supplement: Supplementary file 11 — Source data Fig. 3 [file 44318_2024_149_MOESM11_ESM.zip › Figure 3/3B. SPO merged cropped.tif]

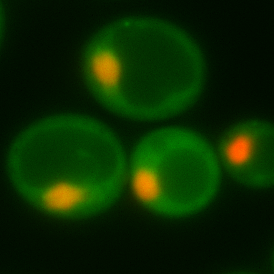

Supplement: Supplementary file 11 — Source data Fig. 3 [file 44318_2024_149_MOESM11_ESM.zip › Figure 3/3B. SPO+glucose merged cropped.tif]

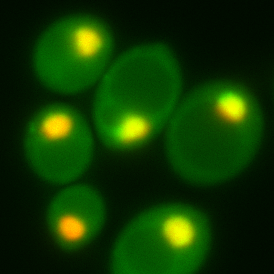

Supplement: Supplementary file 11 — Source data Fig. 3 [file 44318_2024_149_MOESM11_ESM.zip › Figure 3/3B. SPO+rap merged cropped.tif]

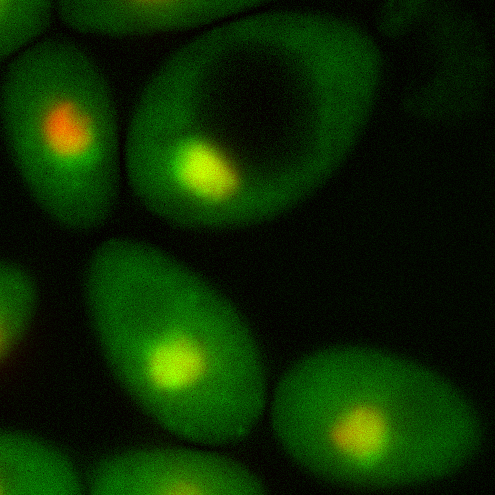

Supplement: Supplementary file 11 — Source data Fig. 3 [file 44318_2024_149_MOESM11_ESM.zip › Figure 3/3C. 1NMPP1 +rapa merged cropped.tif]

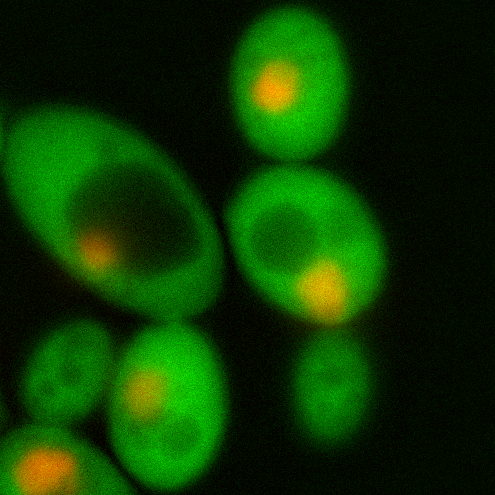

Supplement: Supplementary file 11 — Source data Fig. 3 [file 44318_2024_149_MOESM11_ESM.zip › Figure 3/3C. 1NMPP1 merged cropped.tif]

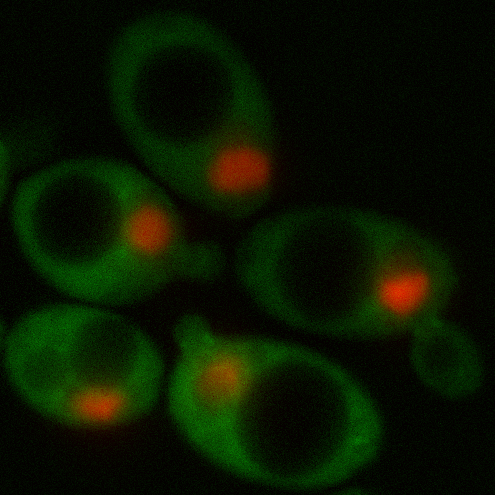

Supplement: Supplementary file 11 — Source data Fig. 3 [file 44318_2024_149_MOESM11_ESM.zip › Figure 3/3C. NT merged cropped.tif]

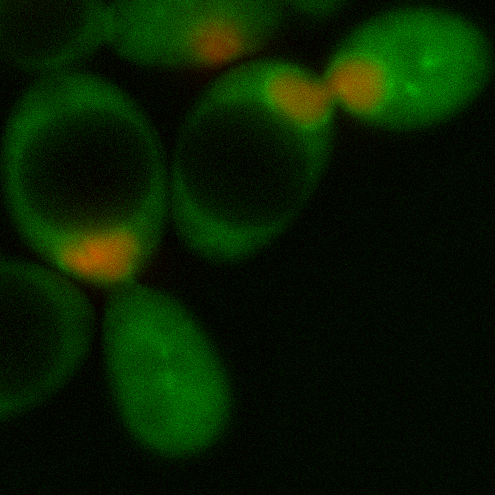

Supplement: Supplementary file 11 — Source data Fig. 3 [file 44318_2024_149_MOESM11_ESM.zip › Figure 3/3C. rapa merged cropped.tif]

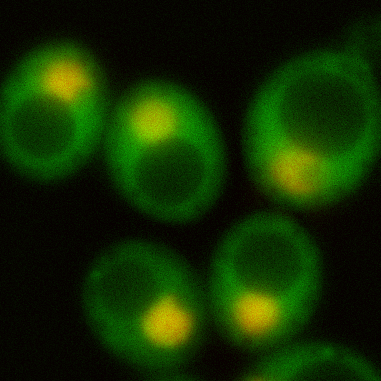

Supplement: Supplementary file 12 — Source data Fig. 4 [file 44318_2024_149_MOESM12_ESM.zip › 4A. 3SA merged cropped.tif]

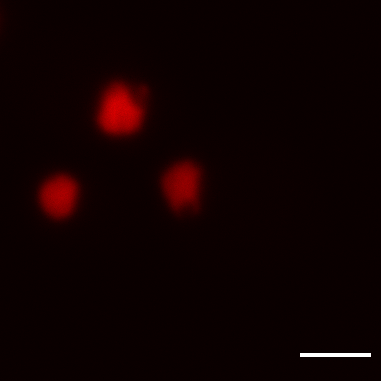

Supplement: Supplementary file 12 — Source data Fig. 4 [file 44318_2024_149_MOESM12_ESM.zip › 4A. WT Htb1-mCherry cropped.tif]

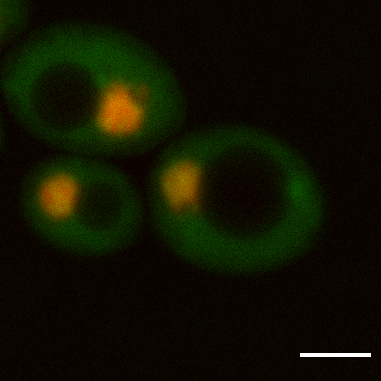

Supplement: Supplementary file 12 — Source data Fig. 4 [file 44318_2024_149_MOESM12_ESM.zip › 4A. WT merged cropped.tif]

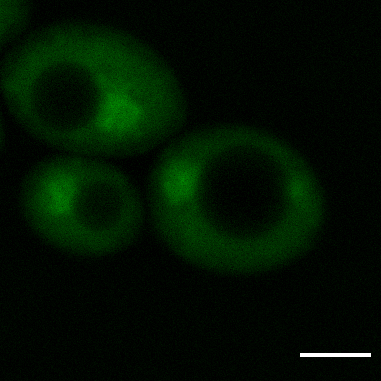

Supplement: Supplementary file 12 — Source data Fig. 4 [file 44318_2024_149_MOESM12_ESM.zip › 4A. WT Rim11-mNG cropped.tif]

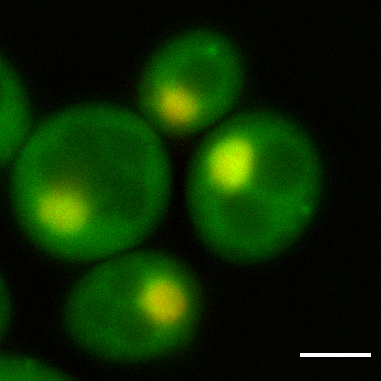

Supplement: Supplementary file 12 — Source data Fig. 4 [file 44318_2024_149_MOESM12_ESM.zip › 4E WT Merged cropped.tif]

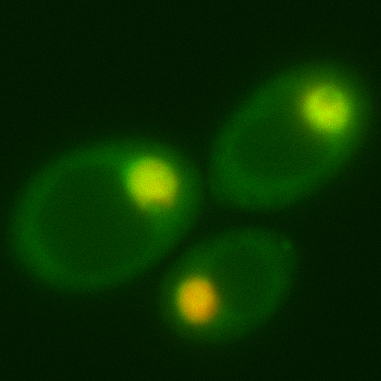

Supplement: Supplementary file 12 — Source data Fig. 4 [file 44318_2024_149_MOESM12_ESM.zip › 4E. mds3D merged cropped.tif]

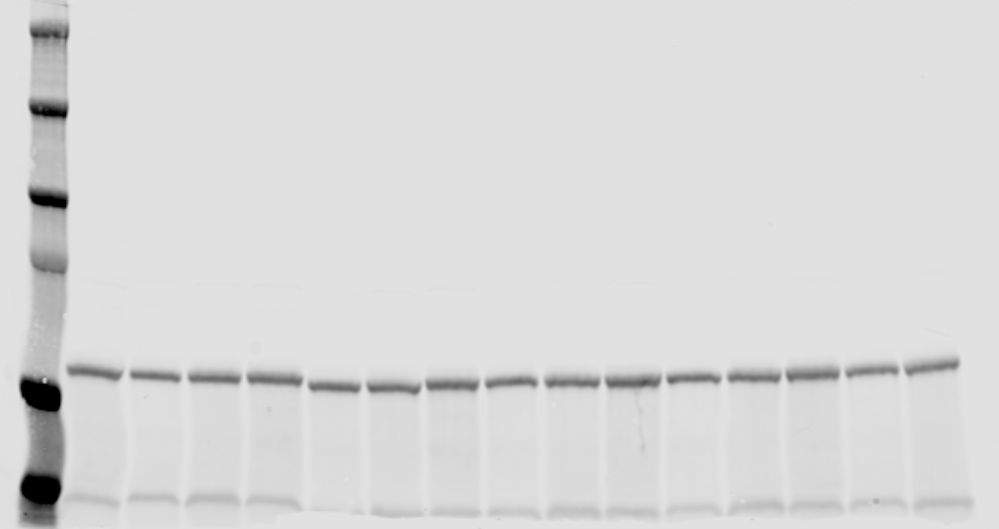

Supplement: Supplementary file 12 — Source data Fig. 4 [file 44318_2024_149_MOESM12_ESM.zip › 4F. Uncropped WB Hxk1.tif]

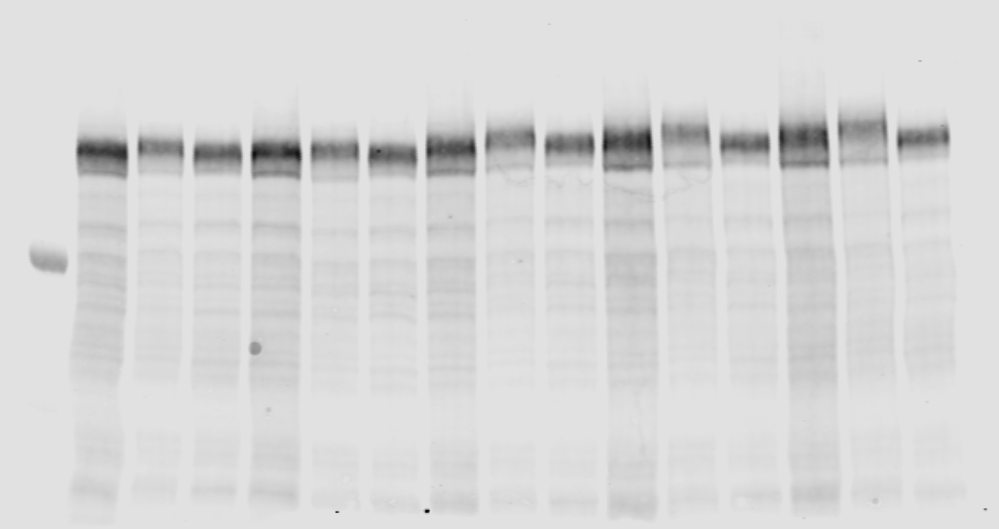

Supplement: Supplementary file 12 — Source data Fig. 4 [file 44318_2024_149_MOESM12_ESM.zip › 4F. Uncropped WB Ume6.tif]

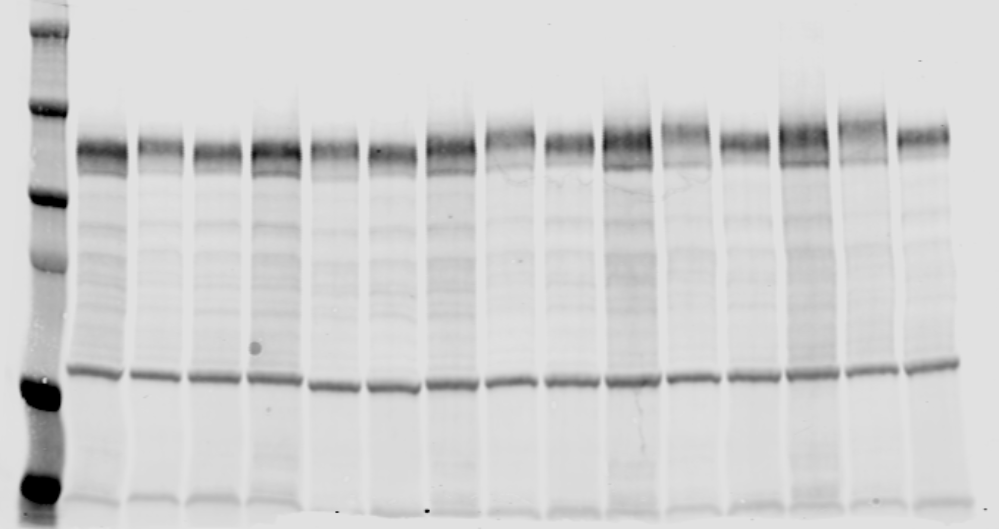

Supplement: Supplementary file 12 — Source data Fig. 4 [file 44318_2024_149_MOESM12_ESM.zip › 4F. Uncropped WB.tif]

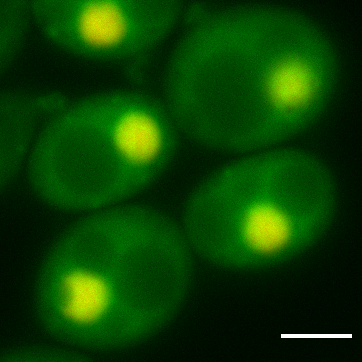

Supplement: Supplementary file 13 — Source data Fig. 5 [file 44318_2024_149_MOESM13_ESM.zip › Figure 5/Figure 5A/Rim11-mNG WT/merged cropped.tif]

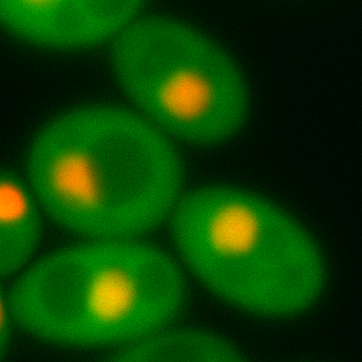

Supplement: Supplementary file 13 — Source data Fig. 5 [file 44318_2024_149_MOESM13_ESM.zip › Figure 5/Figure 5A/Rim11-mNG Y199F/merged cropped.tif]

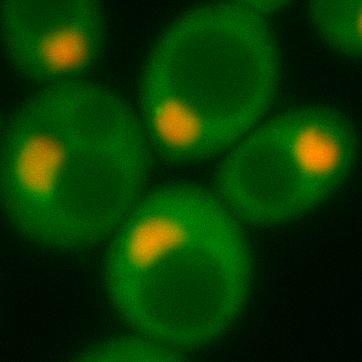

Supplement: Supplementary file 13 — Source data Fig. 5 [file 44318_2024_149_MOESM13_ESM.zip › Figure 5/Figure 5B/Rim11-mNG ime1-delete/merged cropped.tif]

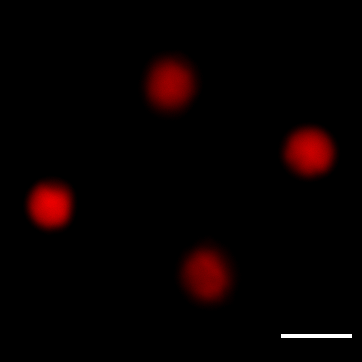

Supplement: Supplementary file 13 — Source data Fig. 5 [file 44318_2024_149_MOESM13_ESM.zip › Figure 5/Figure 5B/Rim11-mNG WT/Htb1-mCherry cropped.tif]

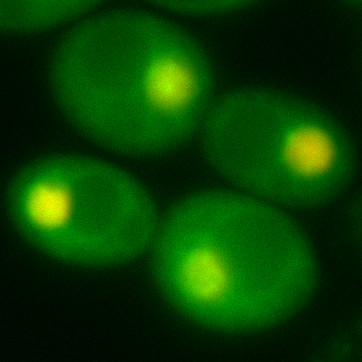

Supplement: Supplementary file 13 — Source data Fig. 5 [file 44318_2024_149_MOESM13_ESM.zip › Figure 5/Figure 5B/Rim11-mNG WT/merged cropped.tif]

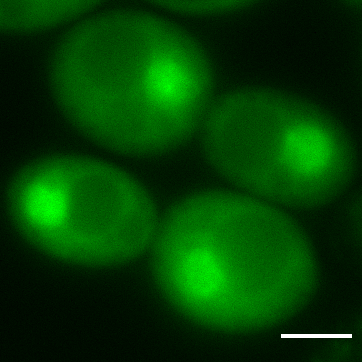

Supplement: Supplementary file 13 — Source data Fig. 5 [file 44318_2024_149_MOESM13_ESM.zip › Figure 5/Figure 5B/Rim11-mNG WT/Rim11-mNG cropped.tif]

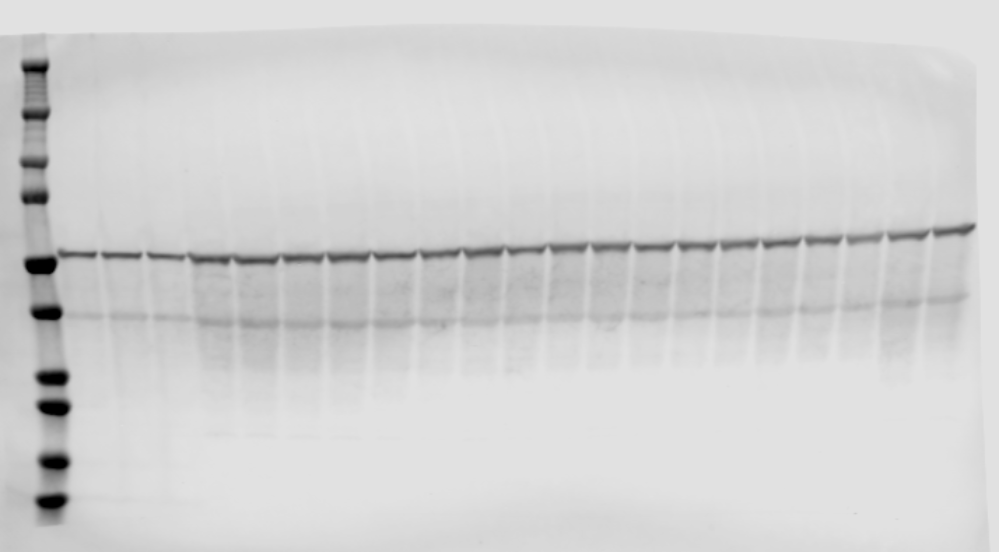

Supplement: Supplementary file 13 — Source data Fig. 5 [file 44318_2024_149_MOESM13_ESM.zip › Figure 5/Figure 5C/Uncropped WB Hxk1.tif]

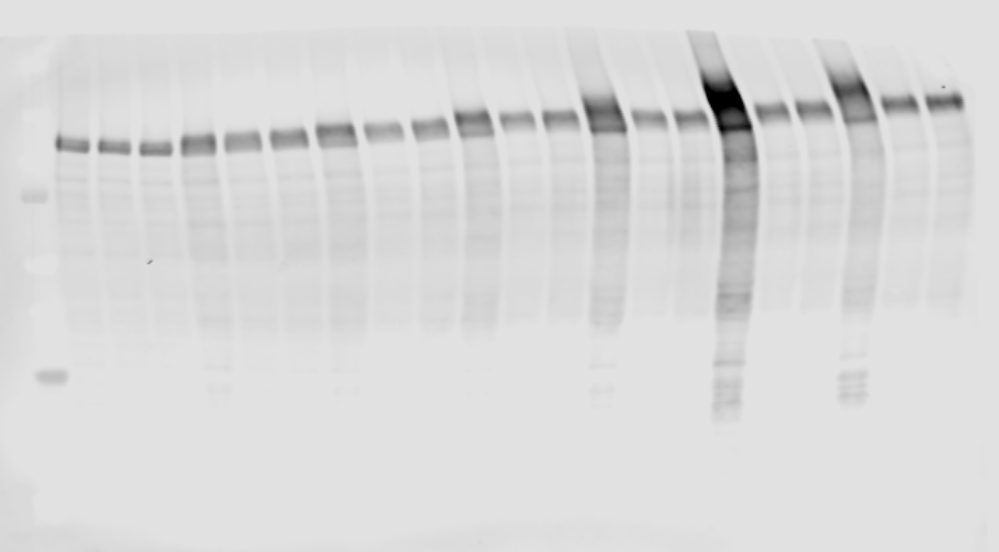

Supplement: Supplementary file 13 — Source data Fig. 5 [file 44318_2024_149_MOESM13_ESM.zip › Figure 5/Figure 5C/Uncropped WB Ume6.tif]

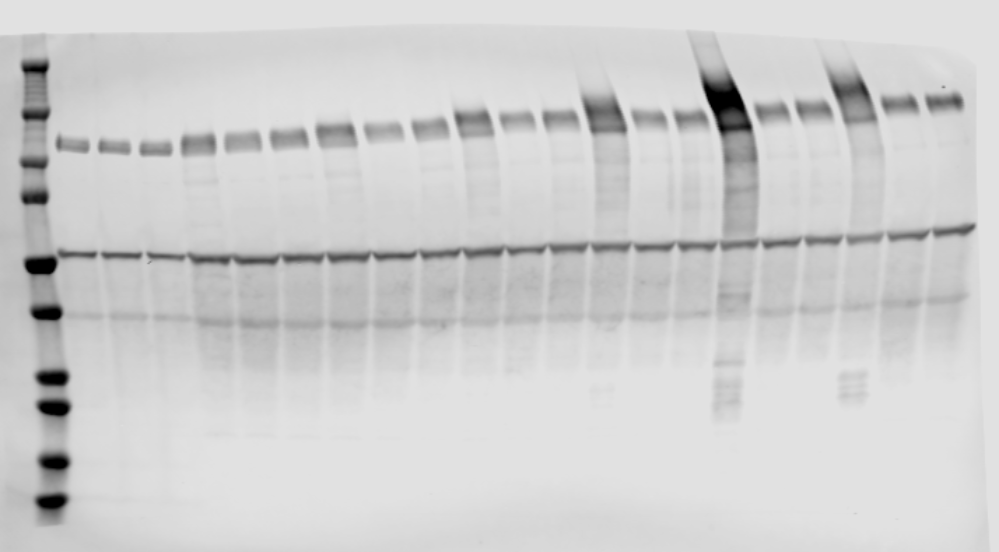

Supplement: Supplementary file 13 — Source data Fig. 5 [file 44318_2024_149_MOESM13_ESM.zip › Figure 5/Figure 5C/Uncropped WB.tif]

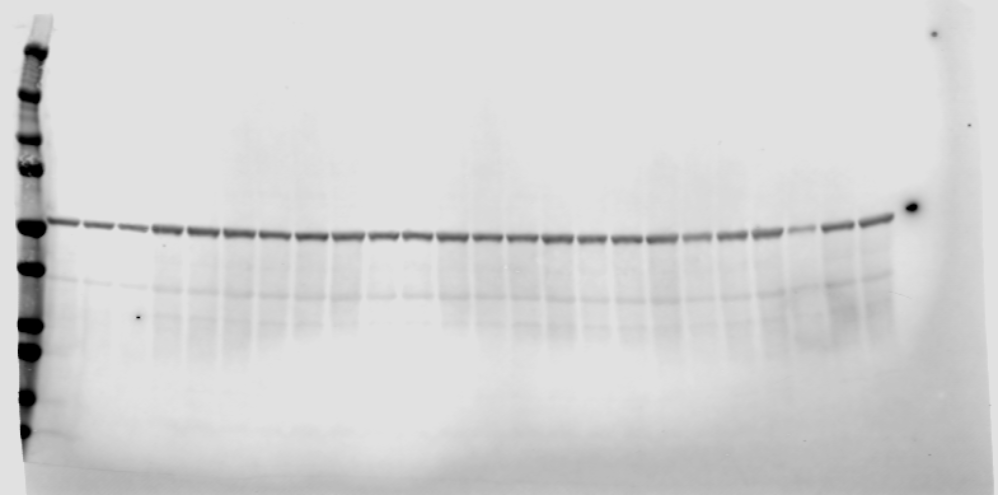

Supplement: Supplementary file 13 — Source data Fig. 5 [file 44318_2024_149_MOESM13_ESM.zip › Figure 5/Figure 5D/Uncropped WB Hxk1.tif]

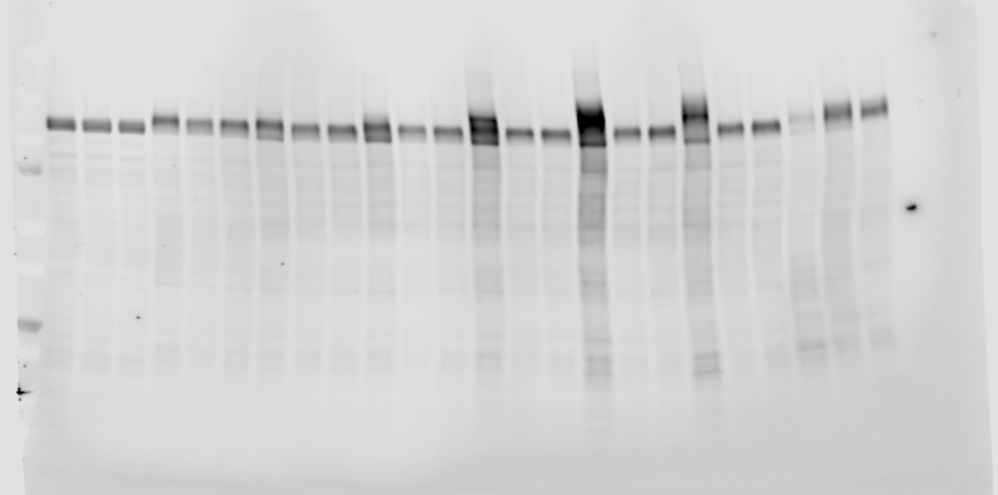

Supplement: Supplementary file 13 — Source data Fig. 5 [file 44318_2024_149_MOESM13_ESM.zip › Figure 5/Figure 5D/Uncropped WB Ume6.tif]

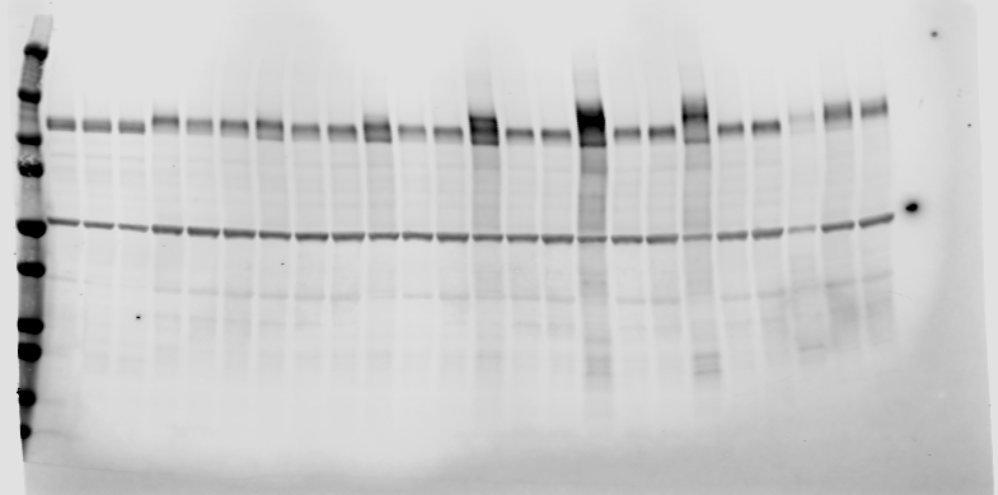

Supplement: Supplementary file 13 — Source data Fig. 5 [file 44318_2024_149_MOESM13_ESM.zip › Figure 5/Figure 5D/Uncropped WB.tif]

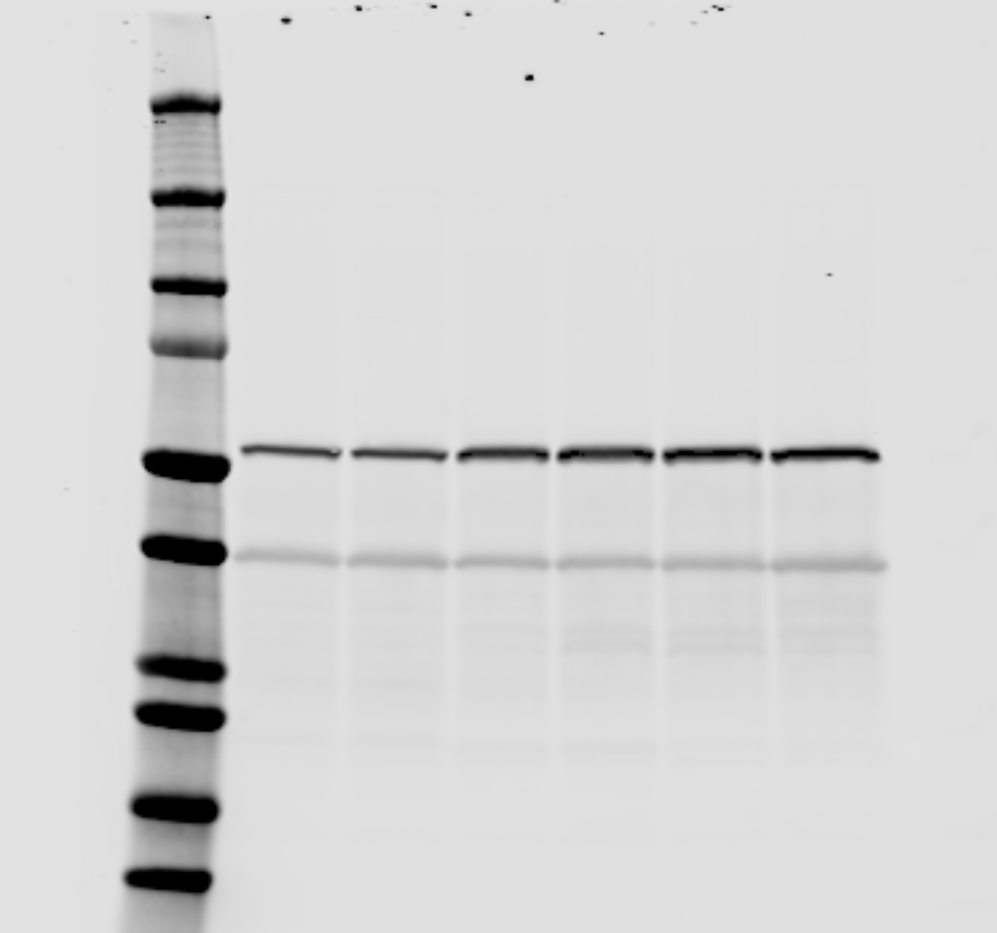

Supplement: Supplementary file 13 — Source data Fig. 5 [file 44318_2024_149_MOESM13_ESM.zip › Figure 5/Figure 5E/Uncropped WB Hxk1.tif]

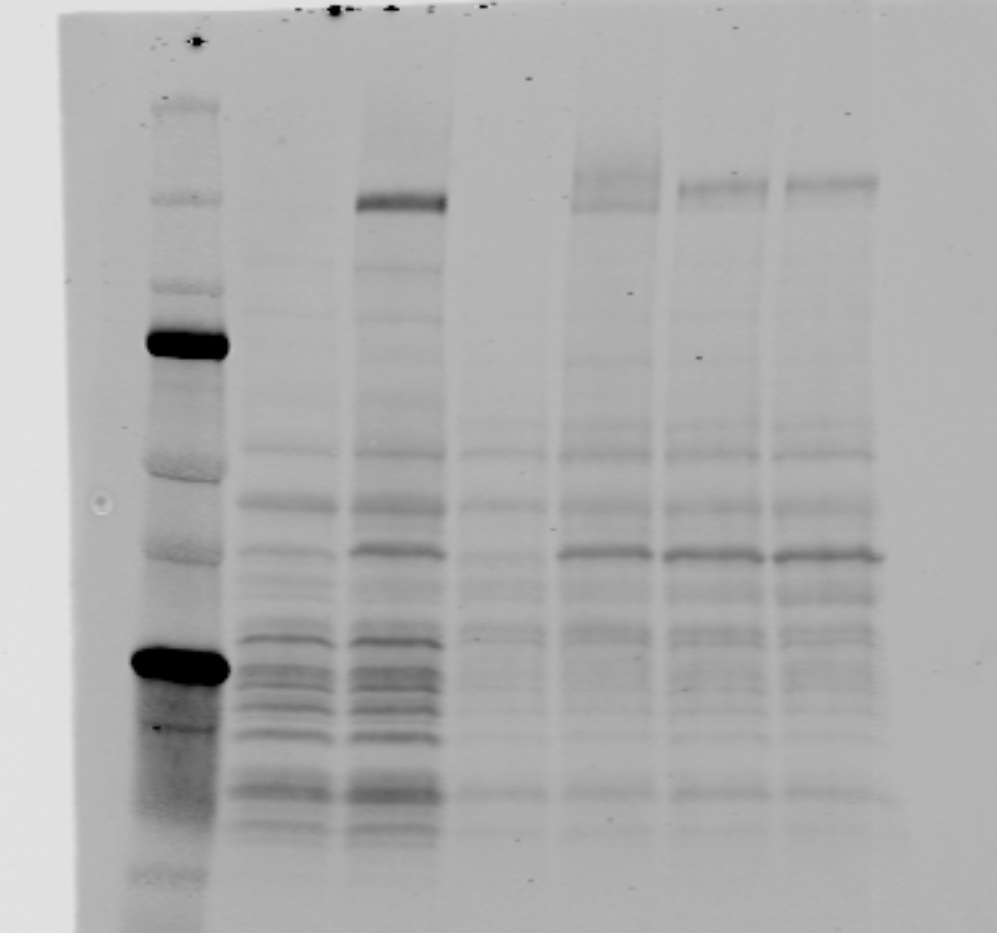

Supplement: Supplementary file 13 — Source data Fig. 5 [file 44318_2024_149_MOESM13_ESM.zip › Figure 5/Figure 5E/Uncropped WB Ume6.tif]

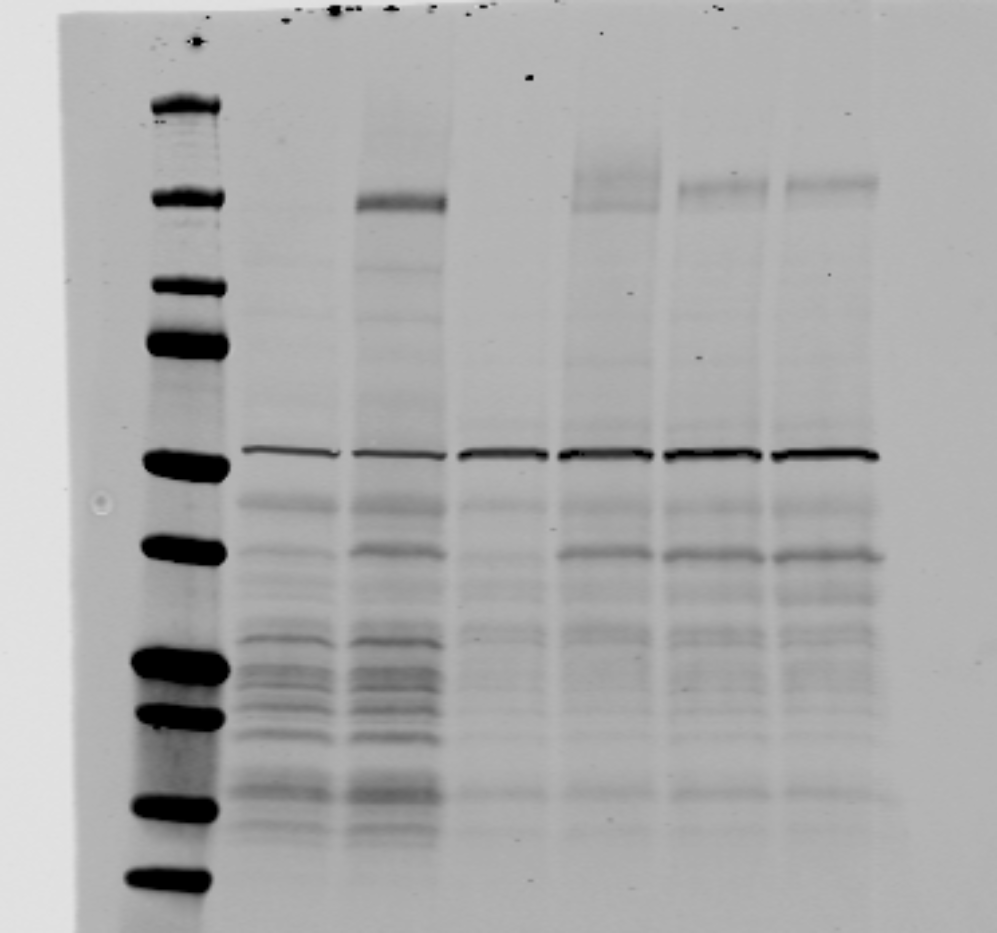

Supplement: Supplementary file 13 — Source data Fig. 5 [file 44318_2024_149_MOESM13_ESM.zip › Figure 5/Figure 5E/Uncropped WB.tif]
