# Supplementary material for: Multi-signal regulation of the GSK-3β homolog Rim11 controls meiosis entry in budding yeast
Source: EMBO J. 2024 Jun 17;43(15):3256–86. doi: 10.1038/s44318-024-00149-7 (PMC11294583; doi:10.1038/s44318-024-00149-7)
Supplement: Supplementary file 16 — Expanded View Figures [file 44318_2024_149_MOESM16_ESM.pdf]

Expanded View Figures

Figure EV1. Rim11 expression and localization dynamics prior and during meiosis.

(**A**) Onset of meiosis in WT (7794) and cells harboring *RIM11* tagged with mNeonGreen (*RIM11*-mNG) (FW10297). Cells were induced to sporulate, and samples were taken at the indicated time points, fixed, and stained with DAPI. Cells that contained two more DAPI masses were considered to have entered meiosis. The error bars represent the mean + SEM of $n = 3$, and least $n = 100$ cells per biological repeat were quantified. (**B**) Nuclear concentrations of Rim11 in exponential growth (exp.) and in cells induced to enter meiosis in SPO and expressing *RIM11*-mNG + *HTB1*-mCh (FW10297). At least $n = 50$ cells per condition were quantified. The error bars represent the mean+SD. (**C**) *RIM11* expression determined by RT-qPCR in WT cells (FW1511) grown until the exponential phase (exp.) and cells induced to enter meiosis. Samples were normalized to the time point 0 h. $n = 2$ biological repeats were performed. The *RIM11* signals were normalized over *ACT1*. (**D**) Quantification of whole-cell concentrations of Rim11-mNG using live-cell imaging setup using the strain described in (**A**). Indicated are the mean traces of cells that entered meiosis (meiosis) and cells that did not enter meiosis (no meiosis). Shown are the Rim11-mNG cytoplasmic concentrations.

 © The Author(s)

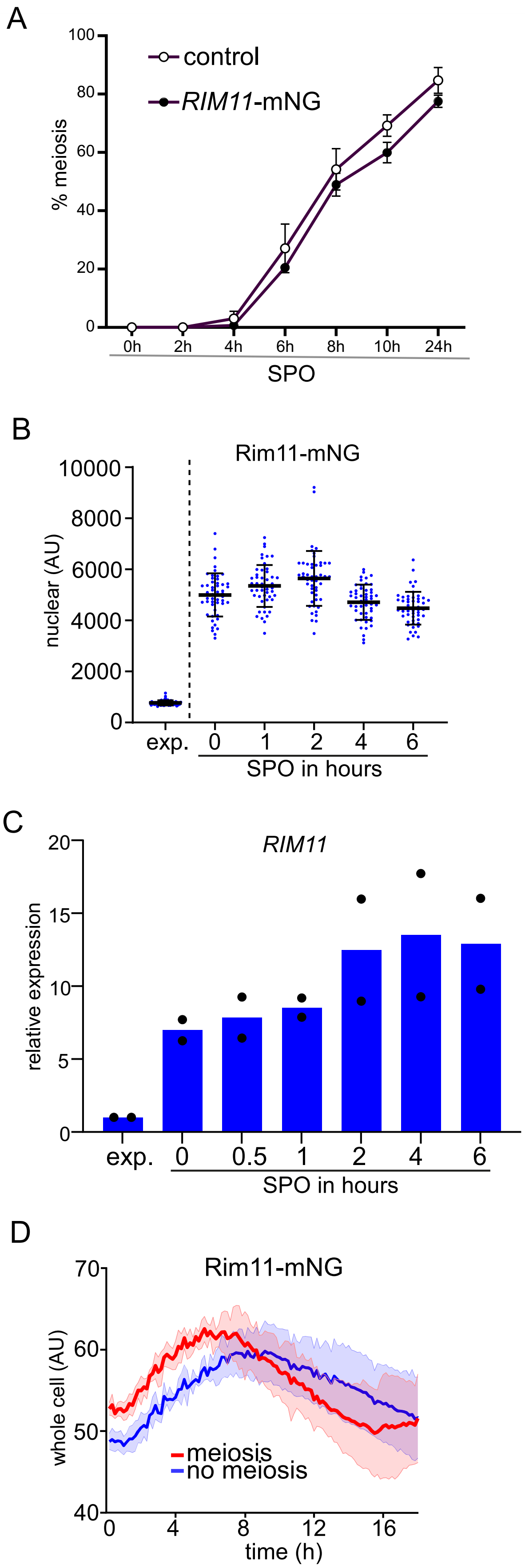
A
100
80
60
40
20
0
% meiosis
control
RIM11-mNG
0h
2h
4h
6h
8h
10h
24h
SPO
B
Rim11-mNG
10000
8000
6000
4000
2000
0
nuclear (AU)
exp.
0
1
2
4
6
SPO in hours
C
RIM11
20
15
10
5
0
relative expression
exp.
0
0.5
1
2
4
6
SPO in hours
D
Rim11-mNG
70
60
50
40
whole cell (AU)
meiosis
no meiosis
0
4
8
12
16
time (h)

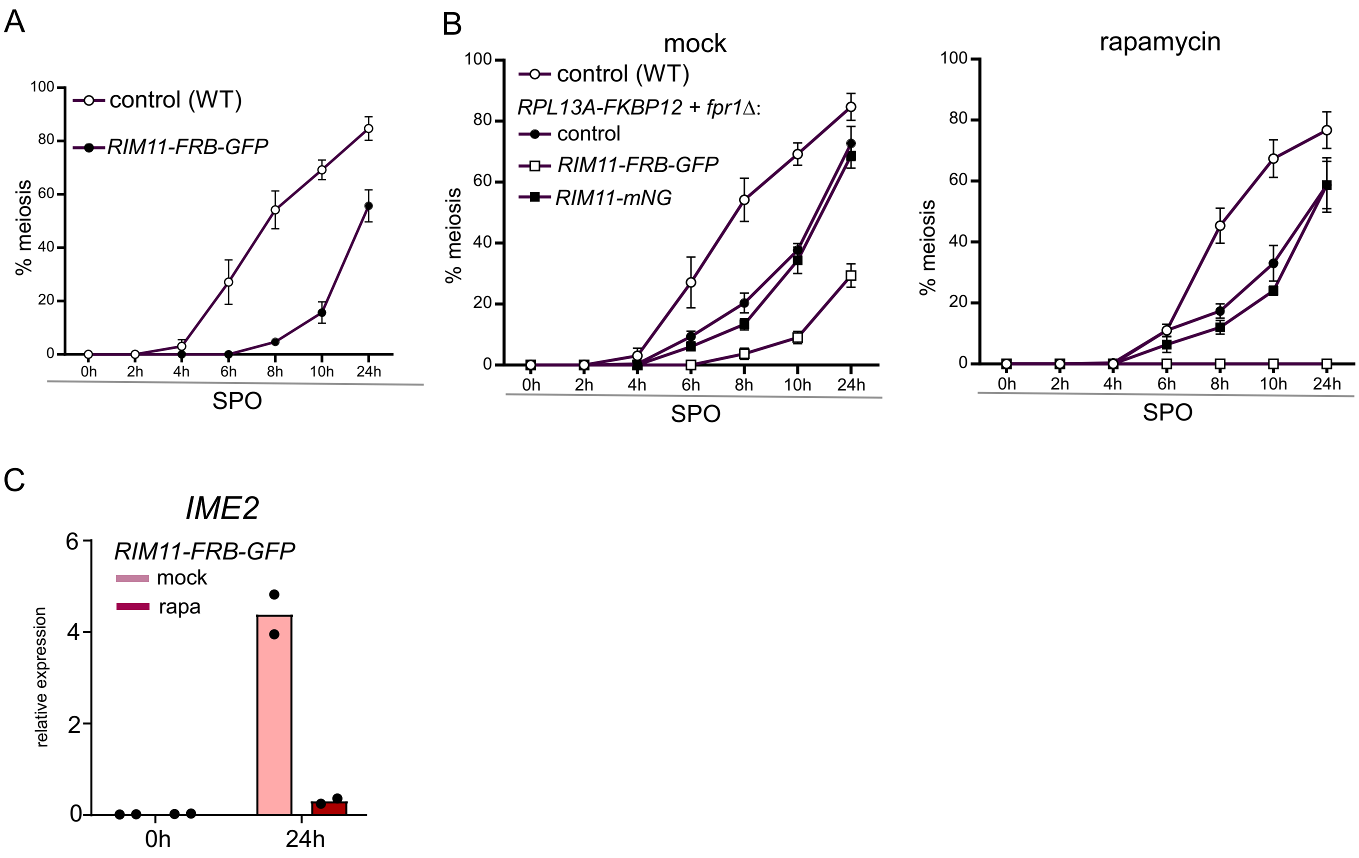

Figure EV2. Rim11 nuclear localization is required for meiosis.

(**A**) Onset of meiosis in WT (7794) and *RIM11-FRB-GFP* (FW11208). Cells were induced to sporulate, and samples were taken at the indicated time points, fixed, and stained with DAPI. Cells that contained two more DAPI masses were considered to have entered meiosis. The error bars represent the mean + SEM of $n = 3$, and at least $n = 100$ cells per biological repeat were quantified. (**B**) Onset of meiosis in WT cells (FW1511), and in cells harboring *RPL13A-FKBP12* and *fpr1Δ* (FW11257) by itself or together with *RIM11-FRB-GFP* (FW11124) or *RIM11-mNG* (FW11126). Cells were mock-treated or untreated with rapamycin at 0 h in SPO. The mean and SEM of 3 independent repeats is shown. (**C**) *IME2* expression was determined in *RIM11-FRB-GFP* (FW11124) after 24 h in SPO in mock and rapamycin-treated cells.

 © The Author(s)

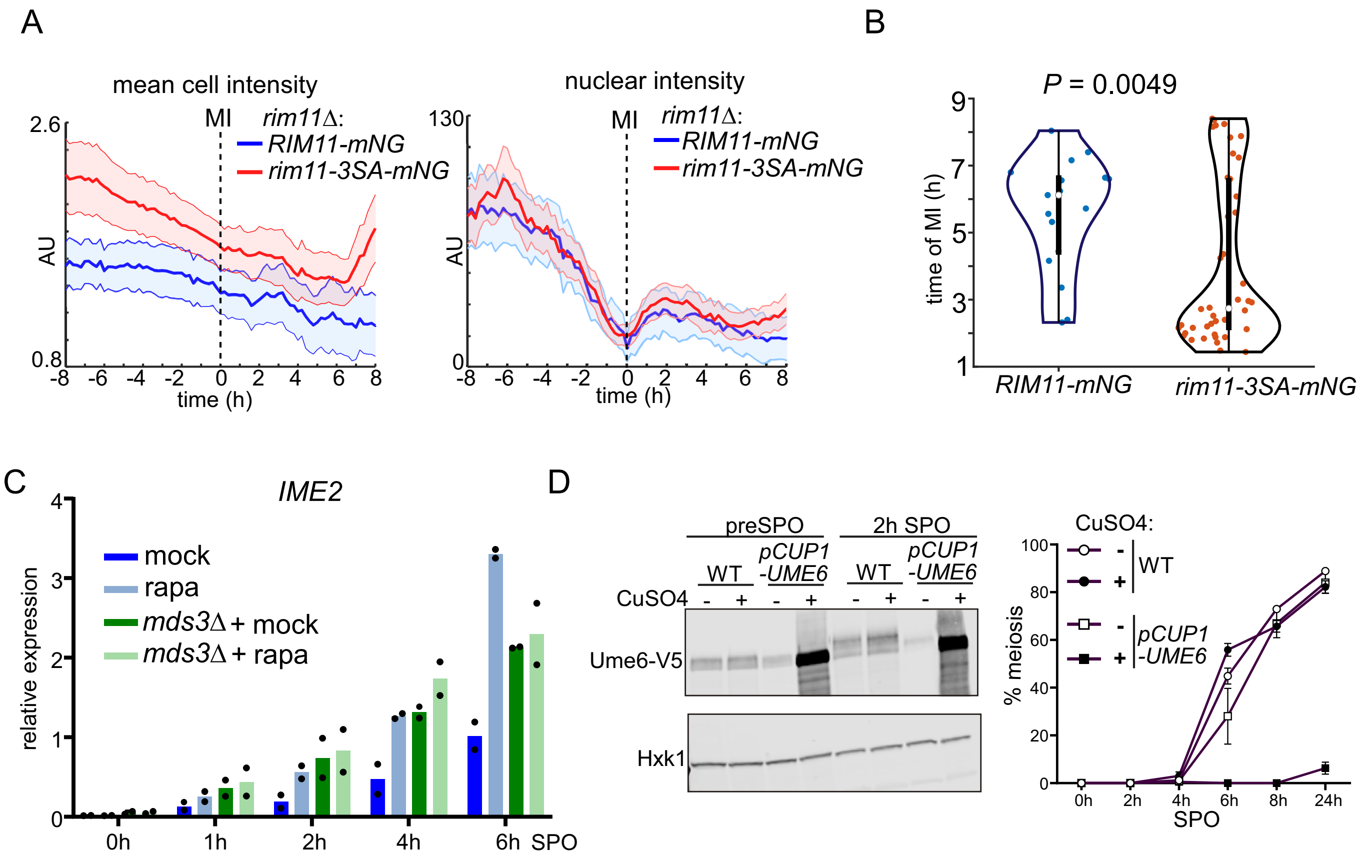

Figure EV3. PKA and TORC1 control Rim11 via distinct mechanisms.

(**A**) Quantification of live-cell imaging of Rim11-mNG and rim11-3SA-mNG. Shown are whole-cell concentration (left) and nuclear intensity (right). Cells harboring *rim11Δ* expressing a single-copy integration plasmid harboring *RIM11-mNG* and *rim11-3SA-mNG* (FW10776 and FW10778). Time points were aligned according to the MI division. At least $n = 500$ cells were quantified. The bold colored line represents the mean, and the 95% intervals are highlighted by the thin colored lines. (**B**) The same experiment as in A but showing the timing of meiosis in Rim11-mNG and rim11-3SA-mNG. $N = 17$ (Rim11-mNG) and $n = 52$ (rim11-3SA-mNG) cells were quantified. The minima and maxima represent the whole distribution range, the center dot the median of the distribution, the bounds of the box represents limits of the 1.5 interquartile range, whiskers represent the whole distribution leaving out the outliers using a 1.5 interquartile outlier criterion. The area around the whisker plot shows the shape of the distribution represented as the kernel density. (**C**) *IME2* expression in cells induced to enter meiosis in WT and *mds3Δ* that were either untreated or treated with rapamycin (FW10297 and FW10718). *IME2* expression signals were normalized over *ACT1*. The mean signals of n = 2 biological repeats are shown. (**D**) Expression and onset of meiosis in *UME6-V5* (FW1208) and *pCUP1-UME6-V5* (FW10562). Cells were grown till the presporulation medium, treated with CuSO4 or untreated, shifted to SPO. Samples were taken at the indicated time points for western blot and DAPI staining. Membranes were probed with anti-V5 antibodies and Hxk1 antibodies as a loading control (left). Cells that contained two more DAPI masses were considered to have entered meiosis. The error bars represent the mean + SEM of $n = 3$, and at least $n = 100$ cells per biological repeat were quantified (right).

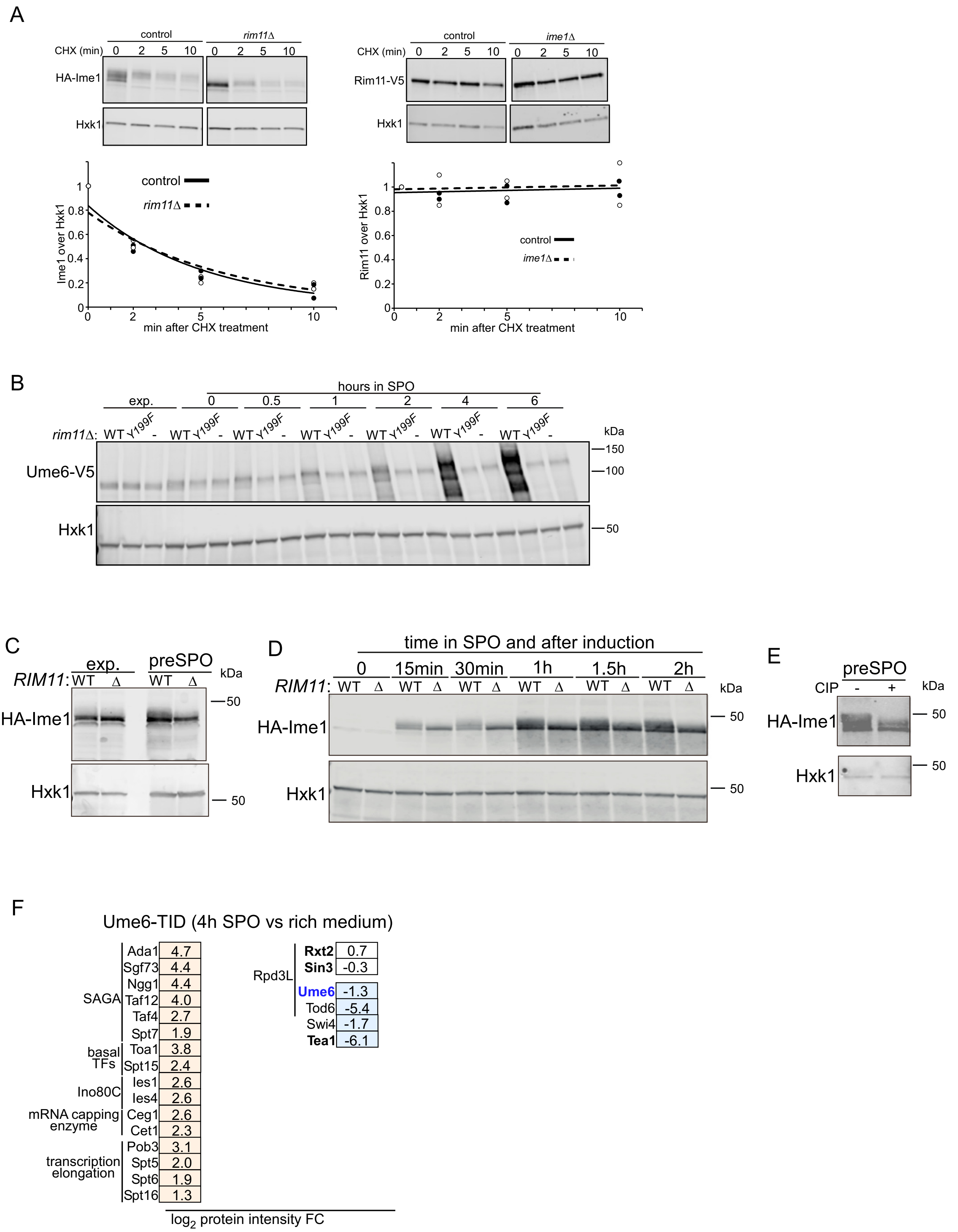
A
control
rim11Δ
CHX (min) 0 2 5 10 0 2 5 10
HA-Ime1
Hxk1
control
ime1Δ
CHX (min) 0 2 5 10 0 2 5 10
Rim11-V5
Hxk1
control
rim11Δ
Ime1 over Hxk1
min after CHX treatment
control
ime1Δ
Rim11 over Hxk1
min after CHX treatment
B
hours in SPO
exp. 0 0.5 1 2 4 6
rim11Δ: WT Y199F -
Ume6-V5
Hxk1
kDa
150
100
50
C
exp. preSPO
RIM11: WT Δ WT Δ
HA-Ime1
Hxk1
kDa
50
D
time in SPO and after induction
0 15min 30min 1h 1.5h 2h
RIM11: WT Δ WT Δ WT Δ WT Δ WT Δ WT Δ
HA-Ime1
Hxk1
kDa
50
E
preSPO
CIP - +
HA-Ime1
Hxk1
kDa
50
F
Ume6-TID (4h SPO vs rich medium)
SAGA
Ada1 4.7
Sgf73 4.4
Ngg1 4.4
Taf12 4.0
Taf4 2.7
Spt7 1.9
basal TFs
Toa1 3.8
Spt15 2.4
Ino80C
Ies1 2.6
Ies4 2.6
mRNA capping enzyme
Ceg1 2.6
Cet1 2.3
transcription elongation
Pob3 3.1
Spt5 2.0
Spt6 1.9
Spt16 1.3
Rpd3L
Rxt2 0.7
Sin3 -0.3
Ume6 -1.3
Tod6 -5.4
Swi4 -1.7
Tea1 -6.1
log2 protein intensity FC

 © The Author(s)

Figure EV4. Ime1 is required for Rim11-directed Ume6 phosphorylation.

(**A**) Relative protein stability of Ime1 and Rim11 in control and in *rim11Δ* or *ime1Δ* cells, respectively. *pCUP-HA-IME1* (control and *rim11Δ*, FW2444 and FW10373) or *RIM11-V5* (control and *ime1Δ*, FW10446 and FW11682) cells were induced to enter meiosis. At 2 h in SPO Ime1 expression was induced with CuSO4. At 4 h cells were treated with cycloheximide (CHX). Samples were taken at the indicated time points for western blotting. Membranes were probed with anti-HA or anti-V5 antibodies. As a loading control Hxk1 was used. Relative quantification with respect Hxk1 and the 0-hour time point is shown for $n = 2$ biological repeats. (**B**) Ume6-V5 expression and migration as determined by western blotting in *rim11Δ* expressing an integration plasmid harboring *RIM11-mNG* or *rim11-Y199F-mNG* cells induced to enter meiosis (FW11186, FW11184, FW10033). Membranes were probes with anti-V5 antibodies and Hxk1 antibodies. (**C–E**) Ime1 expression and migration in exponential growth, and in cells induced to enter meiosis. Ime1 tagged with HA was expressed from the *CUP1* promoter and induced in WT and *rim11Δ* cells (FW2444, FW10373). Samples were taken at the indicated time points, and probed with anti-HA antibodies, and Hxk1 antibodies as a control. In E protein extracts from samples grown in presporulation medium were treated with alkaline phosphatase (CIP). (**F**) Proximity labeling using Ume6-TID comparing 4 h SPO to rich medium conditions. Log2 protein intensity fold change (FC) comparing 4 h SPO to rich medium. Proteins involved in transcription or that are known to interact with Ume6 are shown.

© The Author(s)

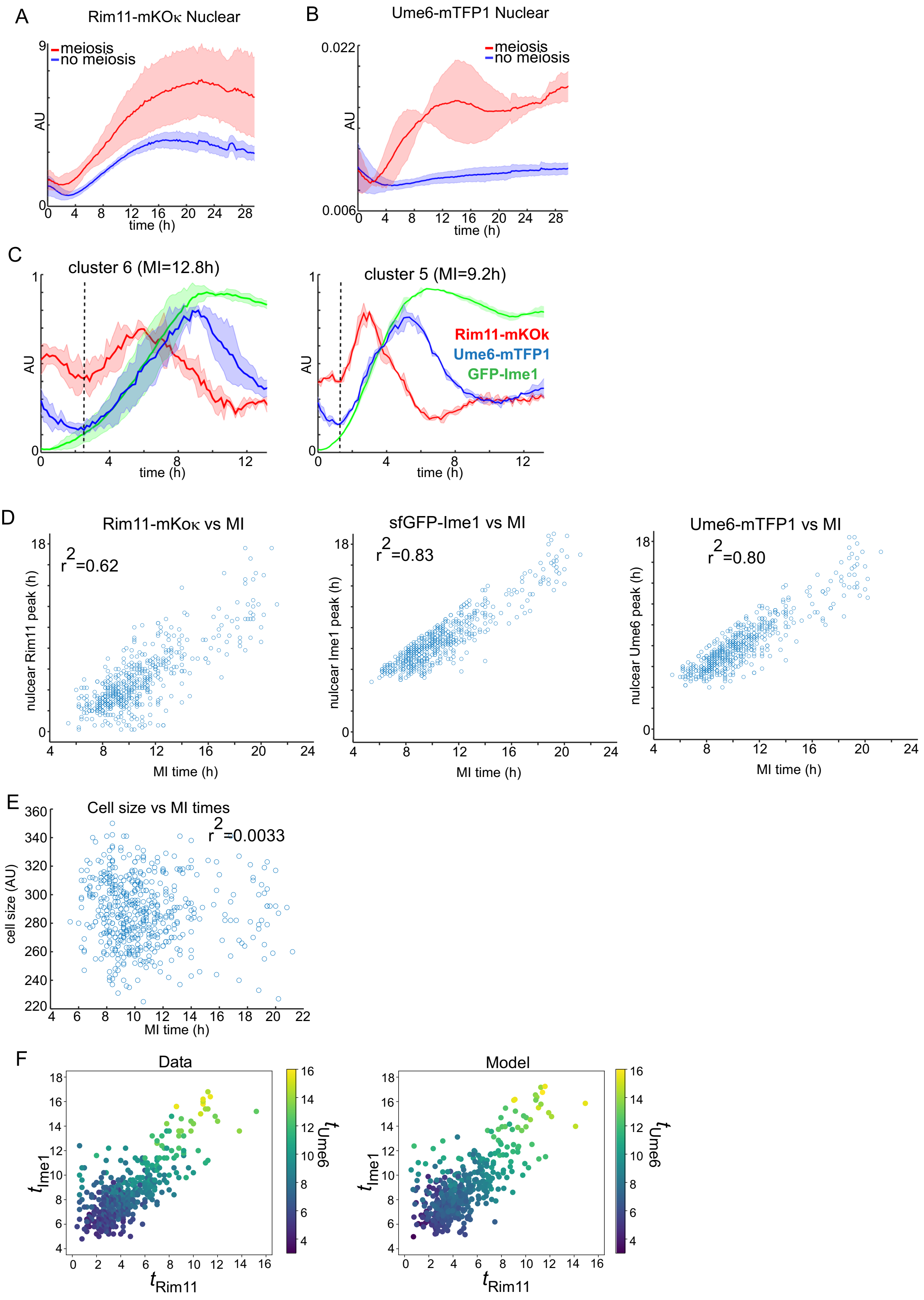
A
Rim11-mKOκ Nuclear
meiosis
no meiosis
AU
time (h)
B
Ume6-mTFP1 Nuclear
meiosis
no meiosis
AU
time (h)
C
cluster 6 (MI=12.8h)
cluster 5 (MI=9.2h)
Rim11-mKOk
Ume6-mTFP1
GFP-Ime1
AU
time (h)
D
Rim11-mKoκ vs MI
sfGFP-Ime1 vs MI
Ume6-mTFP1 vs MI
r2=0.62
r2=0.83
r2=0.80
nulcear Rim11 peak (h)
nulcear Ime1 peak (h)
nulcear Ume6 peak (h)
MI time (h)
E
Cell size vs MI times
r2=0.0033
cell size (AU)
MI time (h)
F
Data
Model
tIme1
tRim11
tUme6

 © The Author(s)

◀ **Figure EV5. Single-cell analysis and modeling reveals the timing dynamics Rim11, Ime1, Ume6 and meiosis.**

Live-cell imaging of the dynamics of Rim11-mKOκ, sfGFP-Ime1, Ume6-mTFP in meiotic and non-meiotic cells (FW11243). (**A**) Nuclear concentrations of Rim1-mKOκ in meiotic and non-meiotic cells. (**B**) Nuclear concentrations of Rim11-mTFP in meiotic and non-meiotic cells. (**C**) Mean traces of Rim11-mKOκ, sfGFP-Ime1, and Ume6-mTFP nuclear intensity for clusters 6 (left), and 5 (right) described in Fig. 7D. (**D**) Scatter plot showing the single-cell data comparing the timing of Rim11-mKOκ peak versus MI (left), sfGFP-Ime1 peak versus MI (middle), Ume6-mTFP peak versus MI (right). (**E**) Scatter plot showing the single-cell data comparing the timing of cell size versus MI. (**F**) Time of Ume6-mTFP peak as a function of sfGFP-Ime1 and Rim11-mKOκ peak timings for the cells that enter meiosis. The left panel shows the results from the time series data, while the right panel illustrates our model predictions. Besides the peak time in Ime1 and Rim11, the model reproduces the amplitudes of the peaks in Ime1 and Rim11 as well as the final value of Rim11 in the single-cell data. The plot contains 461 out of 524 cells—of the remaining 63 cells, most were excluded because the maximal values in Ime1 and Rim11 of the model lie outside the first 18 h after $t = 0$.

© The Author(s)
